# Increased Cerebrospinal Fluid Angiotensin-Converting Enzyme 2 Fragments as a Read-Out of Brain Infection in Patients With COVID-19 Encephalopathy

**DOI:** 10.1093/infdis/jiaf093

**Published:** 2025-02-26

**Authors:** Matthew P Lennol, María-Salud García-Ayllón, Carlos Avilés-Granados, Chiara Trasciatti, Chiara Tolassi, Virginia Quaresima, Davide Arici, Viviana Cristillo, Irene Volonghi, Francesca Caprioli, Valeria De Giuli, Sara Mariotto, Sergio Ferrari, Gianluigi Zanusso, Nicholas J Ashton, Henrik Zetterberg, Kaj Blennow, Alessandro Padovani, Andrea Pilotto, Javier Sáez-Valero

**Affiliations:** Instituto de Neurociencias de Alicante, Universidad Miguel Hernández-Consejo Superior de Investigaciones Científicas (CSIC), San Juan de Alicante, Spain; Centro de Investigación Biomédica en Red Sobre Enfermedades Neurodegenerativas, San Juan de Alicante, Spain; Instituto de Neurociencias de Alicante, Universidad Miguel Hernández-Consejo Superior de Investigaciones Científicas (CSIC), San Juan de Alicante, Spain; Centro de Investigación Biomédica en Red Sobre Enfermedades Neurodegenerativas, San Juan de Alicante, Spain; Unidad de Investigación, Hospital General Universitario de Elche, Fundación para el Fomento de la Investigación Sanitaria y Biomédica de la Comunitat Valenciana (FISABIO), Elche, Spain; Instituto de Neurociencias de Alicante, Universidad Miguel Hernández-Consejo Superior de Investigaciones Científicas (CSIC), San Juan de Alicante, Spain; Centro de Investigación Biomédica en Red Sobre Enfermedades Neurodegenerativas, San Juan de Alicante, Spain; Instituto de Investigación Sanitaria y Biomédica de Alicante (ISABIAL), Alicante, Spain; Neurology Unit, Department of Clinical and Experimental Sciences, University of Brescia, Brescia, Italy; Department of Continuity of Care and Frailty, Neurology Unit, Azienda Socio Sanitaria Territoriale (ASST) degli Spedali Civili Hospital, Brescia, Italy; Neurobiorepository and Laboratory of Advanced Biological Markers, University of Brescia and ASST Spedali Civili Hospital, Brescia, Italy; Neurology Unit, Department of Clinical and Experimental Sciences, University of Brescia, Brescia, Italy; Department of Continuity of Care and Frailty, Neurology Unit, Azienda Socio Sanitaria Territoriale (ASST) degli Spedali Civili Hospital, Brescia, Italy; Neurobiorepository and Laboratory of Advanced Biological Markers, University of Brescia and ASST Spedali Civili Hospital, Brescia, Italy; Neurology Unit, Department of Clinical and Experimental Sciences, University of Brescia, Brescia, Italy; Department of Continuity of Care and Frailty, Neurology Unit, Azienda Socio Sanitaria Territoriale (ASST) degli Spedali Civili Hospital, Brescia, Italy; Neurobiorepository and Laboratory of Advanced Biological Markers, University of Brescia and ASST Spedali Civili Hospital, Brescia, Italy; Neurology Unit, Department of Clinical and Experimental Sciences, University of Brescia, Brescia, Italy; Department of Continuity of Care and Frailty, Neurology Unit, Azienda Socio Sanitaria Territoriale (ASST) degli Spedali Civili Hospital, Brescia, Italy; Neurobiorepository and Laboratory of Advanced Biological Markers, University of Brescia and ASST Spedali Civili Hospital, Brescia, Italy; Neurology Unit, Department of Clinical and Experimental Sciences, University of Brescia, Brescia, Italy; Department of Continuity of Care and Frailty, Neurology Unit, Azienda Socio Sanitaria Territoriale (ASST) degli Spedali Civili Hospital, Brescia, Italy; Neurobiorepository and Laboratory of Advanced Biological Markers, University of Brescia and ASST Spedali Civili Hospital, Brescia, Italy; Neurology Unit, Department of Clinical and Experimental Sciences, University of Brescia, Brescia, Italy; Department of Continuity of Care and Frailty, Neurology Unit, Azienda Socio Sanitaria Territoriale (ASST) degli Spedali Civili Hospital, Brescia, Italy; Neurobiorepository and Laboratory of Advanced Biological Markers, University of Brescia and ASST Spedali Civili Hospital, Brescia, Italy; Neurology Unit, Istituti Ospedalieri, ASST Cremona, Cremona, Italy; Neurology Unit, Istituti Ospedalieri, ASST Cremona, Cremona, Italy; Neurology Unit, Department of Neurosciences, Biomedicine and Movement Sciences, University of Verona, Verona, Italy; Neurology Unit, Department of Neurosciences, Biomedicine and Movement Sciences, University of Verona, Verona, Italy; Department of Neuroscience, Biomedicine, and Movement Sciences, University of Verona, Verona, Italy; Department of Psychiatry and Neurochemistry, Institute of Neuroscience and Physiology, The Sahlgrenska Academy, University of Gothenburg, Gothenburg, Sweden; Institute of Psychiatry, Psychology, and Neuroscience, Maurice Wohl Clinical Neuroscience Institute, King’s College London, London, United Kingdom; Centre for Age-Related Medicine, Stavanger University Hospital, Stavanger, Norway; National Institute for Health and Care Research Biomedical Research Centre for Mental Health and Biomedical Research Unit for Dementia, South London and Maudsley National Health Service Foundation, London, United Kingdom; Banner Sun Health Research Institute, Sun City, Arizona, USA; Department of Psychiatry and Neurochemistry, Institute of Neuroscience and Physiology, The Sahlgrenska Academy, University of Gothenburg, Gothenburg, Sweden; Clinical Neurochemistry Laboratory, Sahlgrenska University Hospital, Mölndal, Sweden; Department of Neurodegenerative Disease, Institute of Neurology, University College London, London, United Kingdom; UK Dementia Research Institute, University College London, London, United Kingdom; Hong Kong Center for Neurodegenerative Diseases, Hong Kong, China; Department of Medicine, School of Medicine and Public Health, University of Wisconsin, Madison, Wisconsin, USA; Department of Psychiatry and Neurochemistry, Institute of Neuroscience and Physiology, The Sahlgrenska Academy, University of Gothenburg, Gothenburg, Sweden; Clinical Neurochemistry Laboratory, Sahlgrenska University Hospital, Mölndal, Sweden; Paris Brain Institute (ICM), Pitié-Salpêtrière Hospital, Sorbonne University, Paris, France; Neurodegenerative Disorder Research Center, Division of Life Sciences and Medicine, and Department of Neurology, Institute on Aging and Brain Disorders, University of Science and Technology of China and First Affiliated Hospital of University of Science and Technology of China (USTC), Hefei, China; Neurology Unit, Department of Clinical and Experimental Sciences, University of Brescia, Brescia, Italy; Department of Continuity of Care and Frailty, Neurology Unit, Azienda Socio Sanitaria Territoriale (ASST) degli Spedali Civili Hospital, Brescia, Italy; Neurobiorepository and Laboratory of Advanced Biological Markers, University of Brescia and ASST Spedali Civili Hospital, Brescia, Italy; Brain Health Center, University of Brescia, Brescia, Italy; Neurology Unit, Department of Clinical and Experimental Sciences, University of Brescia, Brescia, Italy; Department of Continuity of Care and Frailty, Neurology Unit, Azienda Socio Sanitaria Territoriale (ASST) degli Spedali Civili Hospital, Brescia, Italy; Neurobiorepository and Laboratory of Advanced Biological Markers, University of Brescia and ASST Spedali Civili Hospital, Brescia, Italy; Instituto de Neurociencias de Alicante, Universidad Miguel Hernández-Consejo Superior de Investigaciones Científicas (CSIC), San Juan de Alicante, Spain; Centro de Investigación Biomédica en Red Sobre Enfermedades Neurodegenerativas, San Juan de Alicante, Spain; Instituto de Investigación Sanitaria y Biomédica de Alicante (ISABIAL), Alicante, Spain

**Keywords:** ACE2, TMPRSS2, COVID-19, CSF, biomarker

## Abstract

**Background:**

This study assesses the cerebrospinal fluid (CSF) levels of the viral receptor angiotensin-converting enzyme 2 (ACE2) and of the serine protease TMPRSS2 fragments in patients with SARS-CoV-2 infection presenting encephalitis (CoV-Enceph).

**Methods:**

The study included biobanked CSF from 18 CoV-Enceph, 4 subjects with COVID-19 without encephalitis (CoV), 21 with non-COVID-19–related encephalitis (Enceph), and 21 neurologically healthy controls. Participants underwent a standardized assessment for encephalitis. A large subset of samples underwent analysis for an extended panel of CSF neuronal, glial, and inflammatory biomarkers. ACE2 and TMPRSS2 species were determined in the CSF by western blotting.

**Results:**

ACE2 was present in CSF as several species, full-length forms and 2 cleaved fragments of 80 and 85 kDa. CoV-Enceph patients displayed increased CSF levels of full-length species, as well as the 80 kDa fragment, but not the alternative 85 kDa fragment, compared with controls and Enceph patients, characterized by increases of both fragments. Furthermore, TMPRSS2 was increased in the CSF of Enceph patients compared with controls, but not in CoV-Enceph patients. The CoV patients without encephalitis displayed unaltered CSF levels of ACE2 and TMPRSS2 species.

**Conclusions:**

Patients with encephalitis displayed an overall increase in CSF ACE2, probably as a consequence of brain inflammation. The increase of the shortest ACE2 fragment only in CoV-Enceph patients may reflect the enhanced cleavage of the receptor triggered by SARS-CoV-2, thus serving to monitor brain penetrance of the virus associated with the rare encephalitis complication. TMPRSS2 changes in the CSF appeared related to inflammation, but not with SARS-CoV-2 infection.

## Teaser

This study demonstrates an increase in a specific CSF ACE2 fragment in patients with SARS-CoV-2 infection presenting encephalitis that was not noticed in COVID-19 without encephalitis or in non-COVID–related encephalitis. This fragment may serve to monitor virus brain penetrance.

## Graphical Abstract

**Graphical Abstract:**
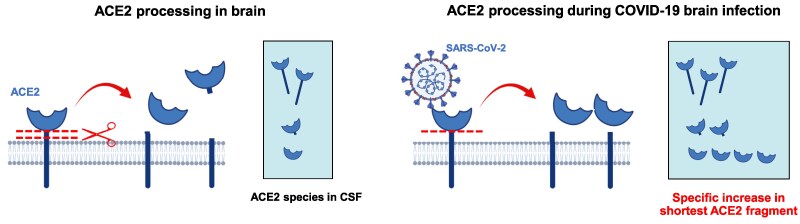

Mild neurological dysfunction is frequent in coronavirus disease 2019 (COVID-19), but severe neurologic disorders can also occur [1]. Indeed, severe acute respiratory syndrome coronavirus 2 (SARS-CoV-2) has been reported to show a capacity for invading the human brain [2]. However, the extent of viral brain infection in COVID-19 patients, and whether neurological symptoms are a primary consequence of SARS-CoV-2 infection or mediated by the neuroinflammatory response, remain unclear [1, 3]. Only very few neurological cases showed threshold amplification in the brain [4, 5]. Most single and multicenter studies indicated that polymerase chain reaction (PCR) for SARS-CoV-2 was negative in the cerebrospinal fluid (CSF) of COVID-19 patients with and without encephalopathy or encephalitis [6, 7]. Thus, it is unclear whether the rare cases of COVID-19–related encephalitis and the subtle neurological symptoms observed are a consequence of neuroinflammation or brain damage directly induced by viral infection [3, 8].

SARS-CoV-2 infects human cells through the binding of viral spike (S) protein to the cellular receptor angiotensin-converting enzyme 2 (ACE2) for entry, and the transmembrane protease serine 2 (TMPRSS2) for S-protein priming. Both ACE2 and TMPRSS2 are proteins present in the human and mouse brain cells [9, 10], and it has been demonstrated that human neurons are a target for SARS-CoV-2 [11]. SARS-CoV-2 infection and replication through the brain also occurs in susceptible transgenic K18-hACE2 mice [12]. Furthermore, neuronal infection can be prevented by blocking ACE2 with antibodies [2].

ACE2 is a type I transmembrane glycoprotein with a short hydrophobic intracellular C-terminus [13]. The protein is cleaved from the plasma membrane by ADAM17 and ADAM10 (sheddases belonging to the “a disintegrin and metalloprotease” family) through constitutive and regulated shedding [14], generating at least 2 large ectodomain fragments [15]. Interestingly, only 1 of the cleaved fragments appears to be regulable by stimulating ADAM17 activity [15]. Despite physiological cleavage, membrane-ACE2 shedding also occurs after SARS-CoV-2 S-protein binding [16]. Recently, we have reported the existence of different species of ACE2 in human fluids that comprise truncated and proteolytically unprocessed full-length forms [17, 18]. Interestingly, in infected humans, during the acute phase of the disease, only the shorter fragment was marginally higher in the plasma of patients with COVID-19 when compared with nondisease controls, whilst the larger fragment remained unaltered, suggesting that only a particular ACE2 fragment could serve as read-out of the processing subsequent to virus entry [17].

TMPRSS2 is a type II transmembrane protein [19] that is expressed as a single chain zymogen that undergoes autoproteolytic cleavage at the ectodomain to acquire proteolytic activity [20]. The cleaved active protease fragment either remains linked to the prodomain via an interdomain disulfide bond or is shed, resulting in a membrane-bound fraction and a cleaved fragment [21]. Circulating TMPRSS2 has been found in human and mouse serum [20, 22, 23]. However, these studies did not address which TMPRSS2 species are present in circulation. To our knowledge, the presence of TMPRSS2 in CSF has not been addressed by any previous studies.

Here, we aimed to determine the levels of ACE2 and TMPRSS2 in the CSF of patients with mild SARS-CoV-2 infection without major neurological symptoms (CoV) and patients severely affected with encephalitis (CoV-Enceph). We also analyzed whether levels of the ACE2 and TMPRSS2 species are differentially affected in patients with encephalitis unrelated to SARS-CoV-2 infection (Enceph). The main objective of the study was to disentangle the relationship between SARS-CoV-2 infection, inflammation-related changes, and ACE2 species in humans. We sought to assess whether the determination of CSF ACE2 serves as a read-out of brain infection in COVID-19 patients.

## METHODS

### Patient Cohorts

Samples and data from patients included in this study were from the ASST Spedali Civili and ASST Cremona Hospitals (Italy), including COVID-19 patients consecutively admitted fulfilling criteria for encephalitis (n = 18) according to a full screening protocol [24, 25]. The study was carried out in accordance with the Declaration of Helsinki. The Institutional Ethical Standards Committee on Human Experimentation at Brescia University Hospital, Italy provided approval for the study (ENCOVID study NP 4131, approved on 25 May 2020), which included informed consent from all the participants. Approval was also obtained by the Ethical Committee of the Universidad Miguel Hernandez, Spain (IN.JSV.03.20). Further information about case definition, ethics and a flowchart summarizing cohort selection is available in the Supplementary Material.

Although lumbar puncture is not a habitual procedure for COVID-19 disease, for biomarker comparison a small group of 4 COVID-19 patients who underwent CSF analyses for isolated persistent headache, but not any other neurological symptom or magnetic resonance imaging/electroencephalogram alteration, was included and considered as a negative control for brain COVID-19–related manifestation. Moreover, patients with encephalitis without SARS-CoV-2 infection (Enceph; 12 with autoimmune-mediated encephalitis and 9 with infectious encephalitis), and neurologically healthy controls with isolated headache (Ctrl; n = 7) with available CSF for ACE2/TMPRSS2 analyses were included.

### Encephalitis Assessment and Diagnosis

First-line testing included all commonly recognized causes of encephalitis according to current guidelines and previous publications [7, 24, 26]. For details see Supplementary Material.

### Determination of ACE2 and TMPRSS2 in CSF Samples by Quantitative Fluorescent Western Blotting

ACE2 and TMPRSS2 species were detected by fluorescent-based imaging after sodium dodecyl sulphate-polyacrylamide gel electrophoresis (SDS-PAGE) and western blotting. CSF samples were prepared and ACE2 or TMPRSS2 were detected as described in the Supplementary Material. ACE2 anti-ectodomain antibodies AF933 (R&D Systems) or antibody MAB933 (R&D Systems), or alternatively with the anti-C-terminus ab15348 antibody (Abcam) were used. TMPRSS2 was detected with the ectodomain antibody 14437-1-AP (Proteintech), the anti-C-terminus antibody H00007113-M05 (Abnova), or the anti-N-terminus antibody OAAB04388 (Aviva Systems Biology).

### CSF Biomarkers for Inflammation, Brain Injury, and Neurodegeneration

A subset of 45 patients with CSF available underwent an additional assessment for inflammatory and neuronal/glial markers: cytokine concentrations (including interleukin 6 [IL-6], IL-8, tumor necrosis factor-α [TNF-α], IL-1β); total tau (T-tau) and phosphorylated tau-181; amyloid-β (Aβ) peptides Aβ38, Aβ40, and Aβ42; neurofilament light polypeptide (NfL); soluble triggering receptor expressed on myeloid cells 2 (sTREM2); glial fibrillary acidic protein (GFAP); and YKL-40, as described in the Supplementary Material; values are indicated in Table 1.

**Table 1.** Biomarker Levels of the Subset Patients With Extended Panel of CSF Markers: COVID-19 Patients With Encephalitis, Non–COVID-19 Related Encephalitis, and Neurologically Healthy Controls

	Cov-Enceph	Enceph	Ctrl	*P* Value
Age, y	67.5 (60.0–73.9)	48.0 (28.4–62.5)	42.0 (37.4–44.5)	<.001^a^
Sex, male:female	11:5	12:7	4:6	.214^b^
P-tau 181, pg/mL	47.0 (43.0–48.2)	22.8 (20.4–35.3)	24.8 (17.1–37.7)	.098^a^
T-tau, pg/mL	322 (231–456)	359 (271–530)	165 (130–267)	.02
NfL, pg/mL	1047 (787–1811)	686 (477–2350)	437 (366–457)	.040
Aβ38, pg/mL	1223 (734–2056)	1403 (1012–2072)	1497 (1349–2128)	.760^a^
Aβ40, pg/mL	3281 (2502–4746)	3869 (3636–5624)	4543 (3948–5992)	.492^a^
Aβ42, pg/mL	251 (105–336)	352 (202–545)	331 (260–515)	.461^a^
Aβ42/Aβ40	0.07 (0.05–0.09)	0.07 (0.06–0.09)	0.07 (0.07–0.08)	.537^a^
IL-1β, pg/mL	0.28 (0.28–0.28)	0.28 (0.27–0.28)	0.27 (0.27–0.27)	.001^c, d, e^
IL-6, pg/mL	1.12 (0.87–3.49)	2.12 (0.71–3.20)	1.14 (0.92–2.15)	.832^c^
IL-8, pg/mL	87.5 (53.3–163)	113 (60.0–143)	52.7 (44.1–70.6)	.162^c^
TNF-α, pg/mL	0.17 (0.16–0.23)	0.29 (0.17–2.30)	0.22 (0.17–0.34)	.156^c^
YKL-40, pg/mL	198 (146–239)	197 (149–313)	83.6 (81.1–106)	.001^c, d, e^
sTREM2, pg/mL	3882 (2205–4848)	3349 (1879–4216)	987 (823–1210)	<.001^c, d, e^
GFAP, pg/mL	444 (251–757)	318 (283–582)	138 (107–174)	.004^c, d, e^

Data are median and interquartile range (25th and 75th percentiles).

Abbreviations: Aβ, amyloid-β; Cov-Enceph, patients with COVID-19 and encephalitis; COVID-19, patiens with coronavirus disease 2019; Ctrl, neurologically healthy controls; Enceph, patients with non–COVID-19 related encephalitis; GFAP, glial fibrillary acidic protein; IL, interleukin; NfL, neurofilament light polypeptide; P-tau 181, phosphorylated tau-181; sTREM2, soluble triggering receptor expressed on myeloid cells 2; TNF-α, tumor necrosis factor-α; T-tau, total tau;

^a^ANOVA test.

^b^Bonferroni/Mann-Whitney test.

^c^Kruskal-Wallis post hoc analysis.

^d^Significant comparison Enceph versus Ctrl.

^e^Significant comparison Cov-Enceph versus Ctrl. Note the lack of significant differences in biomarkers between Enceph and Cov-Enceph patients.

### Statistical Analysis

All data were analyzed using SigmaStat (version 3.5, SPSS). The Kolmogorov-Smirnov test was used to analyze the distribution of each variable. ANOVA was used for parametric variables and the Kruskal-Wallis test for nonparametric variables for comparison between groups; for CSF biomarkers comparison the adjusted threshold was set to 0.05/13 biomarkers, thus 0.004. For categorical variables, a χ^2^ test was used. For direct subgroup comparisons, we used the Bonferroni test and Mann-Whitney tests, when appropriate. The matrix correlations were based on Pearson test.

## RESULTS

### Clinical Characteristics of Patients

The study included 64 participants, namely 18 CoV-Enceph, 21 Enceph, 4 COVID-19 patients (CoV), and 21 neurologically healthy controls (Ctrl). Most CoV-Enceph patients exhibited hyperproteinorrachia and mild pleocytosis with no differences between COVID-19 and non–COVID-19 patients, as previously reported [7]. As detailed in the Supplementary Material, all CoV-Enceph had a negative result for infectious and autoimmune encephalitis screening, as well as for SARS-CoV-2 presence in CSF by reverse transcription PCR (RT-PCR).

### CSF ACE2 Species Are Altered in Patients With Encephalitis; Differences Between Patients With Encephalitis With and Without SARS-CoV-2 Infection

**Figure 1.**
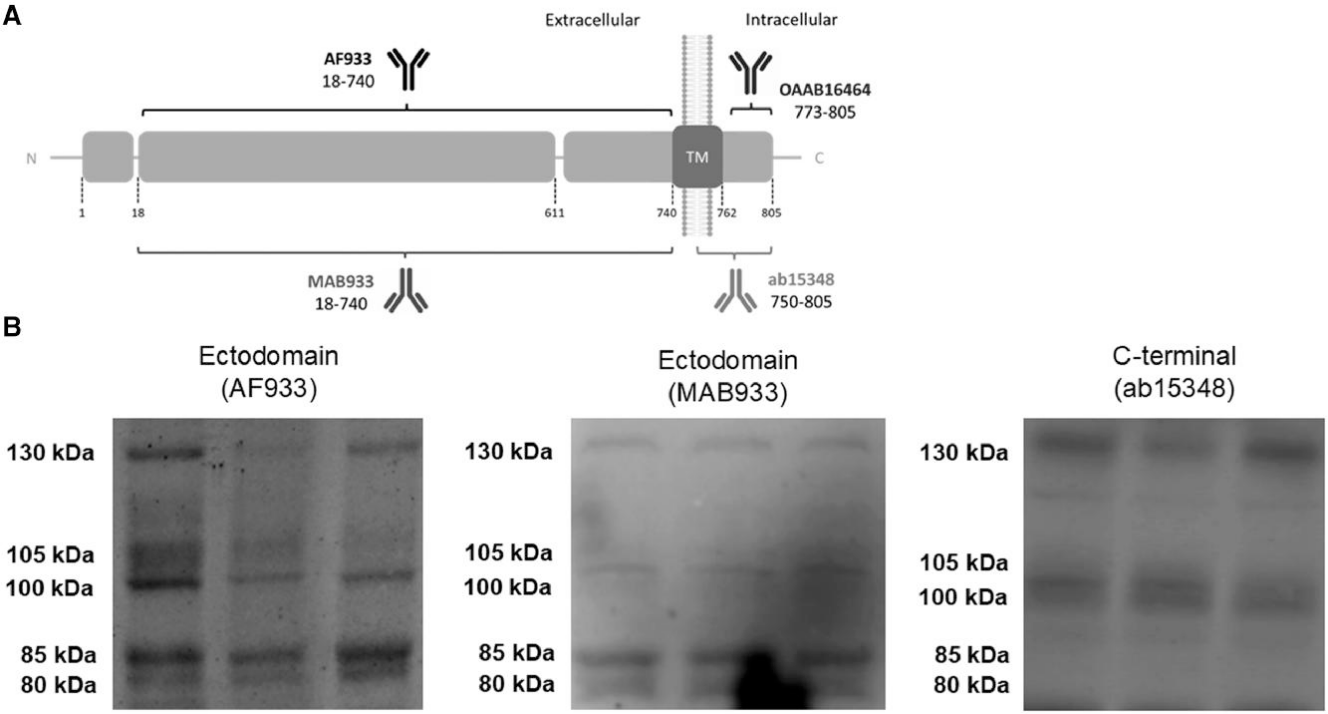
Several ACE2 full-length and fragment species coexist in CSF and result altered in patients with encephalitis with and without SARS-CoV-2 infection. *A*, Schematic representation of ACE2 as a transmembrane type I protein, and of the epitopes recognized by antibodies used in this study (not drawn to scale). ACE2 anti-ectodomain antibodies were AF933 (R&D Systems; goat polyclonal; immunogen, amino acid residues 18–740; 1:200 dilution) and MAB933 (R&D Systems; mouse monoclonal; immunogen, amino acid residues 18–740; 1:500 dilution); anti-C-terminus was antibody ab15348 (Abcam; rabbit polyclonal; immunogen, synthetic peptide corresponding to human ACE2 amino acid residues 788–805; 1:500 dilution). The signal peptide (aa 1–17), carboxypeptidase (aa 18-611), and transmembrane (aa 740–762) domains are represented. The sites of ACE2 shedding with ADAM17 are also indicated (between aa 652–659 and 697–416). The resulting ectodomain fragments are recognized by the AF933 and MAB933 antibodies, but not by the C-terminal antibody ab15348. *B*, Representative blots of ACE2 immunoreactive species in CSF from nondisease controls using different antibodies, as indicated. Abbreviations: aa, amino acid; ACE2, angiotensin-converting enzyme 2; CSF, cerebrospinal fluid; SARS-CoV-2, severe acute respiratory syndrome coronavirus 2; TM, transmembrane.

Because soluble full-length species and fragments of ACE2 have been demonstrated in biological fluids [17], we analyzed CSF samples by western blotting, allowing the separation of individual species, and using ectodomain antibodies (recognizing full-length species and C-terminal truncated fragments), or an antibody raised against the C-terminus of human ACE2 (recognizing only full-length species); see scheme in Figure 1*A*. Analysis revealed a similar banding pattern to that reported for human plasma [17, 27], confirming the existence in the CSF of full-length ACE2 species of approximately 100 and 105 kDa, as well as an approximately 130-kDa species, all of which were immunoreactive to both the ectodomain and C-terminal antibodies (Figure 1*B*). Two additional bands of approximately 80 and 85 kDa were uniquely immunoreactive to the ectodomain antibodies AF933 and MAB933, thus they probably represent cleaved fragments (Figure 1*B*).

**Figure 2.**
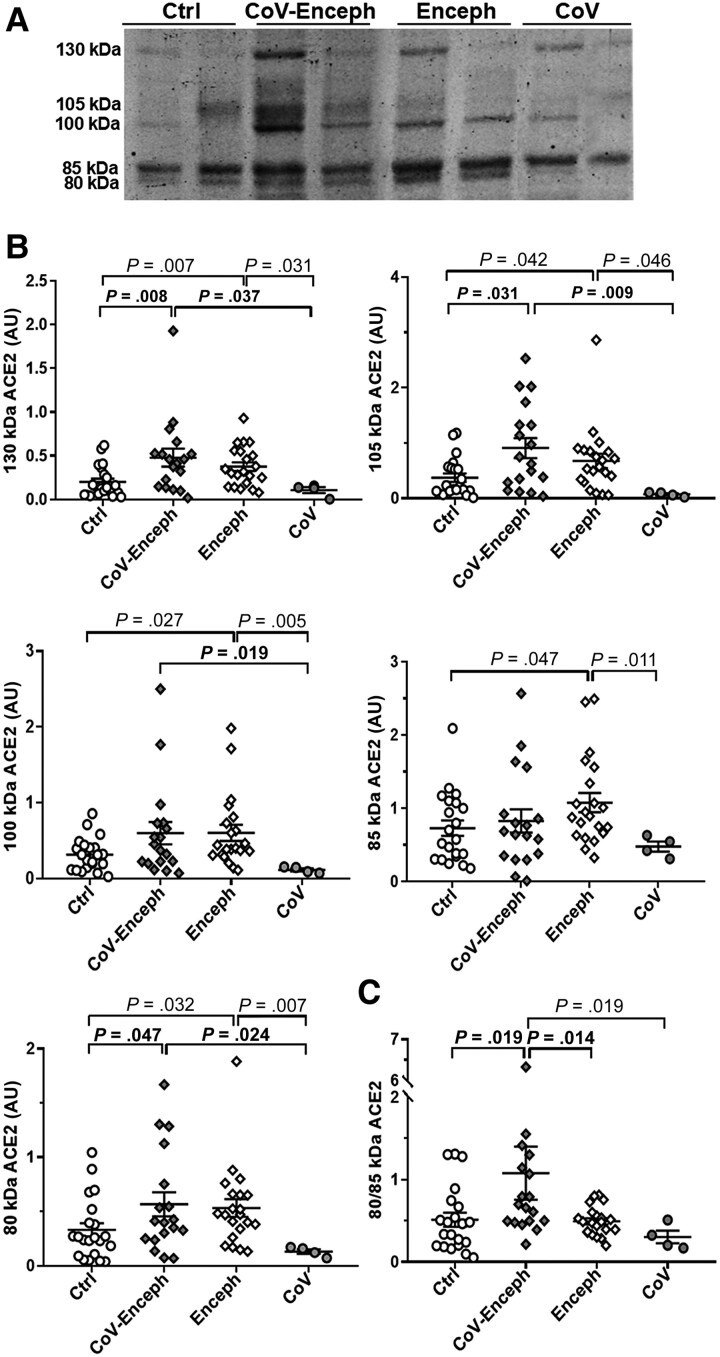
Altered balance of ACE2 fragments is a feature of patients with encephalitis and SARS-CoV-2 infection. *A*, Levels of CSF ACE2 species were determined in neurologically healthy controls (Ctrl), patients with SARS-CoV-2 infection with encephalitis (CoV-Enceph), patients with encephalitis without SARS-CoV-2 infection (Enceph), and patients with SARS-CoV-2 infection without neurological symptoms (CoV). Samples were blotted with the AF933 antibody against the ectodomain of ACE2. *B*, Immunoreactivity levels were determined for the 85- and 80-kDa fragments and for the 130-, 105-, and 100-kDa full-length species. *C*, The relationship between ACE2 fragments, for each sample, are represented by the graph of the quotient obtained by dividing the level of immunoreactivity of the 80 kDa band by the level of immunoreactivity of the 85 kDa band (80/85 kDa). Values are presented as mean ± SEM; *P* values are indicated; significant changes compared with CoV-Enceph group are highlighted in bold. Abbreviations: ACE2, angiotensin-converting enzyme 2; AU, arbitrary unit; CSF, cerebrospinal fluid; SARS-CoV-2, severe acute respiratory syndrome coronavirus 2.

Next, we examined the levels of ACE2 species in the CSF by quantitative fluorescent western blotting resolved with the AF933 antibody, which detected all ACE2 species (Figure 2*A*). CoV-Enceph patients displayed significantly higher levels of the 80-kDa fragment (71%; *P* = .040), but not the 85-kDa fragment, which was unaltered compared with controls (Figure 2*B*). The 130-kDa (139% increase; *P* = .008) and 105-kDa full-length species (144%; *P* = .031) also presented higher levels in CoV-Enceph patients, with a trend to an increase for the 100-kDa species (89%; *P* = .132; Figure 2*B*). Samples from Enceph patients displayed elevated levels for all ACE2 species compared with controls, including the 80-kDa (61%; *P* = .032) and 85-kDa (48%; *P* = .047) fragments, and full-length species, as well as the 130-kDa (88%; *P* = .007), 105-kDa (81%; *P* = .042), and 100-kDa (90%; *P* = .027) species. We did not observe changes in the levels of ACE2 species for CoV patients without major neurological symptoms compared to controls (Figure 2*B*). The finding was statistically significant, despite the low statistical power due to the small number of CoV cases without encephalitis.

ACE2 is cleaved from the plasma membrane resulting in fragments with a molecular mass approximately 20 kDa less than the original full-length species [15, 28]; thus, we cannot discount that part of the 100- and 105-kDa immunoreactive species correspond to cleaved fragments derived from the 130-kDa plasma-membrane ACE2 species. However, we found a good correlation between immunoreactivity levels resolved with the C-terminal antibody ab15348 and values of the same immunoreactive band estimated with the ectodomain antibody AF933 for the 100-kDa (*r* = 0.90; *P* < .001) and 105-kDa (*r* = 0.73; *P* < .001) bands, estimated separately or together (*r* = 0.85; *P* < .001).

As a general statement, an overall increase in ACE2 full-length species would translate to an increase in all the generated fragments, at least through constitutive shedding; however, a shedding mediated by interaction of the receptor with a ligand, such as SARS-CoV-2, could result in generation of a specific fragment. We further evaluated whether 80-kDa fragment shedding increased as a result of SARS-CoV-2 infection, by calculating a ratio for the ACE2 fragments, the 80 kDa/85 kDa ACE2 quotient (Figure 2*C*). Of note, COVID-19 patients with and without manifestation of neurological encephalitis showed a significant difference between the quotient compared to the other groups.

### CSF TMPRSS2 Species Are Altered in Patients With Encephalitis But Are Not Associated With COVID-19

**Figure 3.**
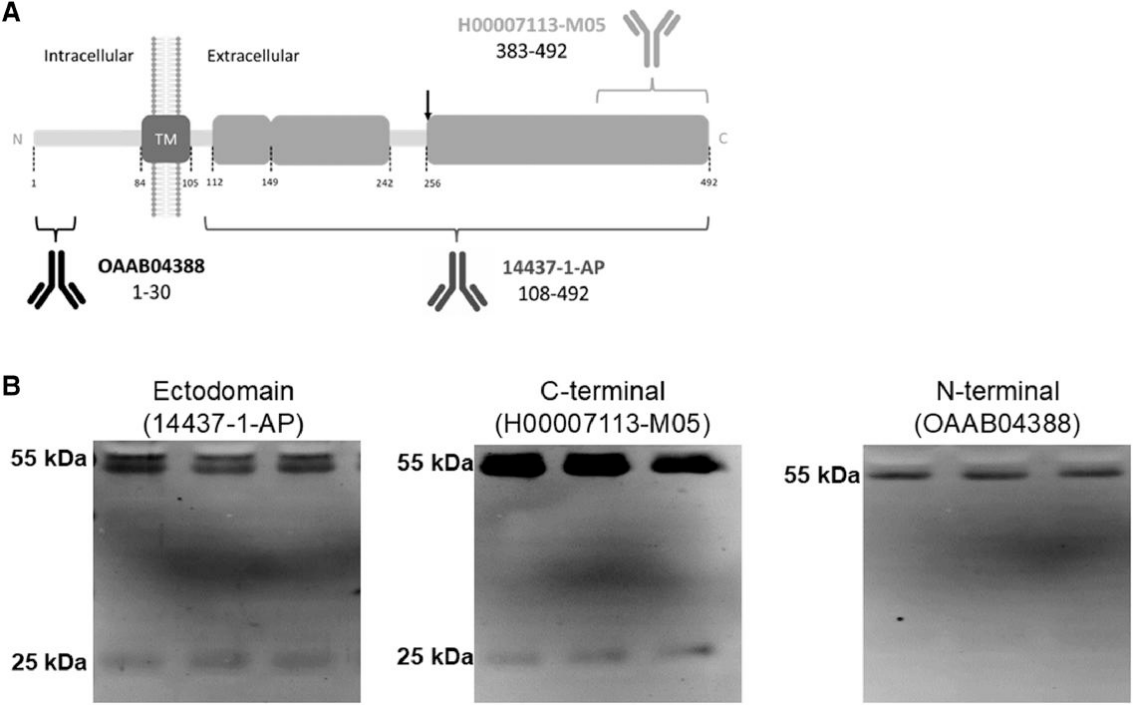
TMPRSS2 full-length and active fragments coexist in CSF and their relative abundances are altered in patients with encephalitis. *A*, Schematic representation of TMPRSS2 as a transmembrane type II protein and of the epitopes recognized by the antibodies used in this study (not drawn to scale). TMPRSS2 was resolved with the ectodomain antibody 14437-1-AP (Proteintech; rabbit polyclonal; immunogen, a recombinant protein containing aa 108–492; 1:1000 dilution), the anti-C-terminus antibody H00007113-M05 (Abnova; mouse monoclonal; immunogen, a recombinant protein containing aa 383–492; 1:500 dilution), or the anti-N-terminus antibody OAAB04388 (Aviva Systems Biology; rabbit polyclonal; immunogen, synthetic peptide between aa 1 and 30; 1:1000 dilution). The site of autoproteolytic cleavage at the ectodomain to acquire proteolytic activity is indicated (aa 255–256). The resulting ectodomain fragment is recognized by the 14437-1-AP and C-terminal antibodies, but not by the N-terminal antibody OAAB04388. The transmembrane (aa 84–105) domain is also indicated. *B*, Representative blots of TMPRSS2 immunoreactive bands present in nondisease CSF samples using different antibodies, as indicated. Abbreviations: aa, amino acid; CSF, cerebrospinal fluid; SARS-CoV-2, severe acute respiratory syndrome coronavirus 2; TM, transmembrane; TMPRSS2, transmembrane protease serine 2.

As mentioned above, the membrane-resident form of TMPRSS2 (a type II transmembrane protein) undergoes autoproteolytic cleavage at the C-terminal ectodomain to generate the protease-active fragment [20], but higher molecular mass species, which may represent full-length forms, have also been found by electrophoresis analysis in cellular media of cells expressing TMPRSS2 [20] and in human semen [29]. Thus, CSF samples were also analyzed by western blotting using either antibodies that recognize the N-terminus, the C-terminal, or the ectodomain of TMPRSS2; see scheme in Figure 3*A*. The evaluation of ectodomain antibodies indicated the existence in the CSF of full-length TMPRSS2 species of approximately 55 kDa, attributed to the zymogen, and minor amounts of a 25-kDa form only immunoreactive to C-terminal antibodies, attributed to the cleaved active fragment (Figure 3*B*).

**Figure 4.**
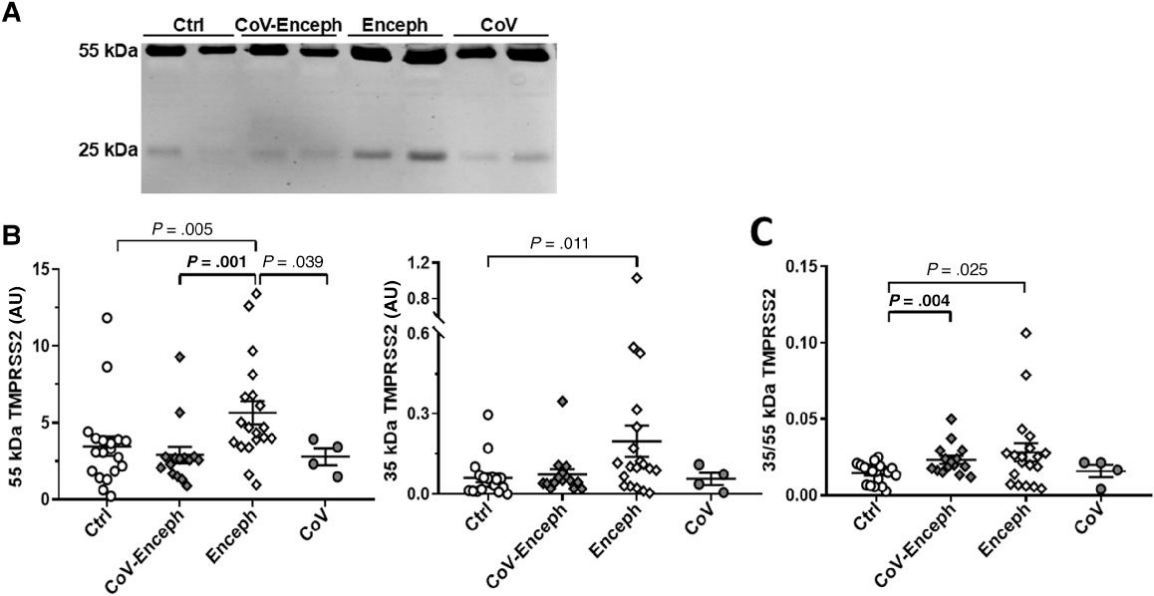
Altered balance of TMPRSS2 active fragment in patients with encephalitis. *A*, CSF samples from neurologically healthy controls (Ctrl), patients with SARS-CoV-2 infection with encephalitis (CoV-Enceph), patients with encephalitis without SARS-CoV-2 infection (Enceph), and patients with SARS-CoV-2 infection without neurological affectation (CoV) were probed with the 14437-1-AP antibody against the ectodomain of TMPRSS2. *B*, Immunoreactivity levels were determined for the 25-kDa fragment and for the 55-kDa full-length species. *C*, The relationship between the fragment and full-length species, for each sample, are represented by the graph of the quotient obtained from the 25-kDa compared to the levels of the 55-kDa TMPRSS2 species (25/55 kDa). Values are presented as mean ± SEM; *P* values are indicated; significant changes compared with CoV-Enceph group are highlighted in bold. Abbreviations: ACE2, angiotensin-converting enzyme 2; AU, arbitrary unit; CSF, cerebrospinal fluid; SARS-CoV-2, severe acute respiratory syndrome coronavirus 2; TMPRSS2, transmembrane protease serine 2.

When we assessed the levels of TMPRSS2 in the CSF resolved with the ectodomain 14437-1-AP antibody (Figure 4*A*), Enceph samples displayed significantly increased levels of both the 25-kDa active species (227%; *P* = .011) and the 55-kDa zymogen (63%; *P* = .005) compared with controls (Figure 4*B*). Both CoV-Enceph and CoV without neurological symptoms showed unaltered levels in TMPRSS2 species when compared with controls (Figure 4*B*). The increased levels of the 55-kDa TMPRSS2 in Enceph patients was also significantly different when compared to the CoV (*P* = .039) or CoV-Enceph (*P* = .001) groups.

The extent of the increase in Enceph patients was different for TMPRSS2 species; the large increase in the 25-kDa fragment resulted in an altered 25 kDa/55 kDa quotient compared with controls (*P* = .025). Of note, the CoV-Enceph group also displayed differences in this 25 kDa/55 kDa quotient compared with controls (*P* = .004), but this resulted from a nonsignificant increase in the 25-kDa fragment (22% increase compared with controls; *P* = .36), while the 55-kDa full-length species tended to decrease, failing to reach significance (17% decrease compared with controls; *P* = .30) (Figure 4*C*).

### Correlations Between CSF Markers, ACE2 Species, and TMPRSS2

The large subset of 45 patients with fully available CSF additional neuronal, amyloid-related, glial, and inflammatory CSF markers are listed in Table 1. Briefly, the encephalitis groups exhibited increased GFAP, sTREM2, YKL-40, and IL-1β compared to controls, with similar findings in the Cov-Enceph and Enceph groups.

**Figure 5.**
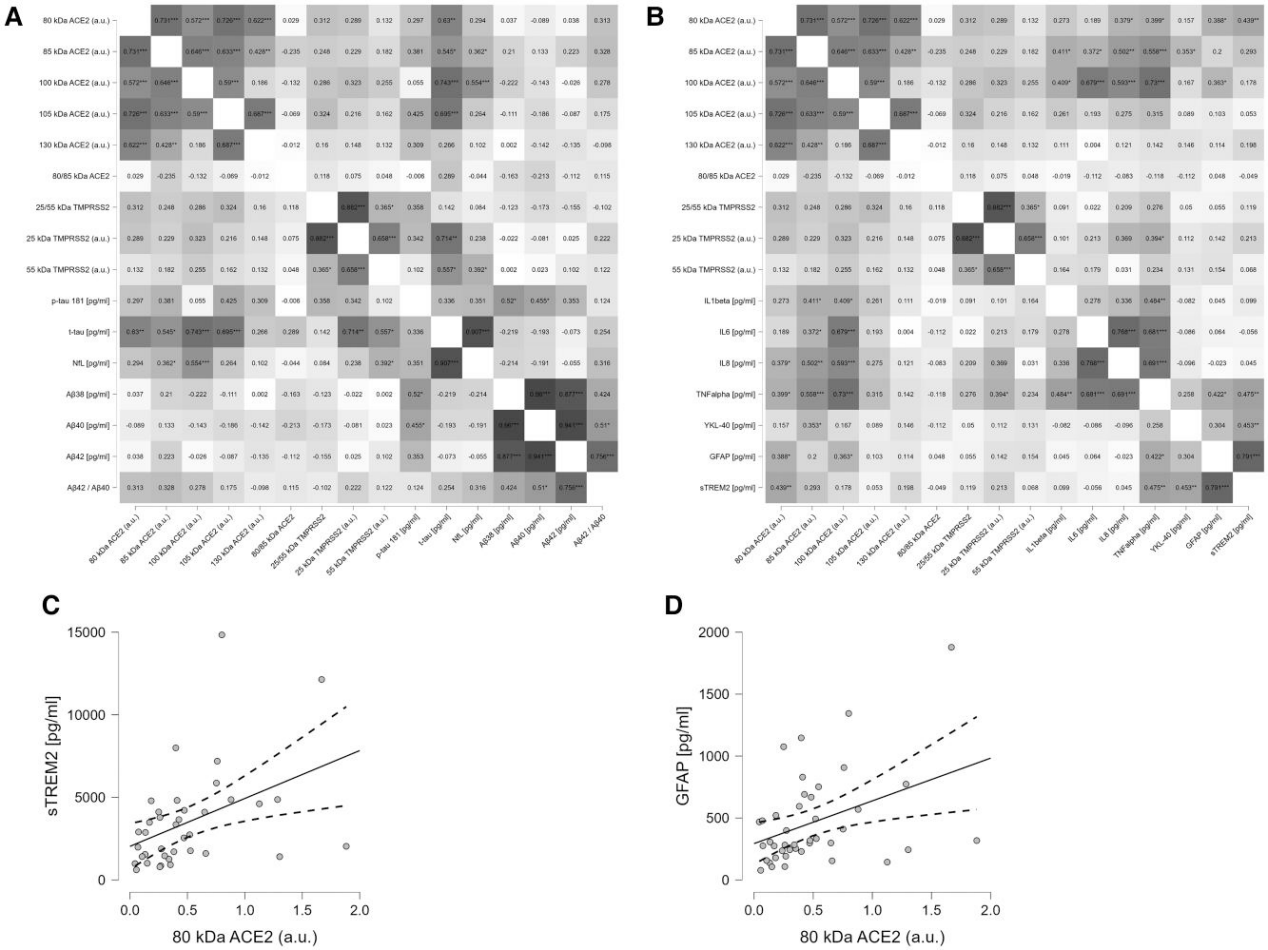
Correlation matrices based on Pearson test for all the determined biomarkers from patients with SARS-CoV-2 infection with encephalitis. *A*, Correlation matrix, corrected for age and sex, of the neurodegeneration markers analyzed in the cohort. *B*, Correlation matrix, corrected for age, sex, and NfL, of the cytokines and glial markers. Matrices show the value of each of the correlation coefficients; significant correlations are indicated: *P < .5, **P < .05, ***P < .001. *C* and *D*, Scatter plot adjusted for age and sex, consistent with the associated correlation matrix, representing correlation of the 80 kDa fragment with (*C*) sTREM2 and (*D*) GFAP (solid line represent the correlation, dashed lines represent the 95% confidence interval). Abbreviations: ACE2, angiotensin-converting enzyme 2; Aβ, amyloid-β; AU, arbitrary unit; GFAP, glial fibrillary acidic protein; IL-1β, interleukin 1β; NfL, neurofilament light polypeptide; P-tau, phosphorylated tau; SARS-CoV-2, severe acute respiratory syndrome coronavirus 2; sTREM2, soluble triggering receptor expressed on myeloid cells 2; TNF-α, tumor necrosis factor-α; T-tau, total tau.

Correlations between ACE2 species, neuronal and glial markers, and cytokine profiles are highlighted in the correlation matrix (Figure 5*A*  and 5*B*). Briefly, several ACE2 and TMPRSS2 species exhibited a strong correlation with T-tau levels, and several of them also correlated with NfL, both of which are considered to be neuronal injury markers. Various ACE2 species, especially 105-kDa ACE2, correlated with inflammatory markers, namely IL-1β, IL-6, and IL-8, also after adjustment for T-tau or NfL levels (ie, independent from neuronal death/dysfunction). No correlations were found between TMPRSS2 fragments and glial markers (sTREM2, YKL-40, GFAP), while the 80-kDa ACE2 fragment correlated weakly, but significantly, with sTREM2 (*r* = 0.439) and GFAP (*r* = 0.388) (Figure 5*C*  and 5*D*).

## DISCUSSION

Our findings show increased levels in a specific ACE2 fragment in the CSF of subjects with encephalitis and COVID-19 that is not present in subjects with encephalitis alone, or subjects with COVID-19 in the absence of severe manifestations. ACE2 full-length species in CSF should reflect the species in the brain, while fragments possibly originate from shedding after interaction with SARS-CoV-2. In a previous report on SARS-CoV-2–infected K18-hACE2 transgenic mice, we identified an early increase in the ACE2 fragment, probably representing the virus entry and subsequent proteolytic processing of the membrane receptor, and a later increase in full-length forms, probably representing tissular ACE2 upregulation as a consequence of massive inflammation [18]. Moreover, of the 2 ACE2 fragments characterized in nonpathological conditions, only the shorter fragment appeared elevated in plasma from mildly affected COVID-19 patients [17]. Similarly, in this study we found a particular increase in the shortest 80-kDa fragment in the CSF of CoV-Enceph patients, without parallel changes in other ACE2 fragments. Therefore, we deduced that particular increases in the shortest ACE2 fragment is a CSF-specific finding in infected subjects and may serve as a read-out to monitor penetrance of the SARS-CoV-2 in the brain leading to COVID-19–related encephalitis. Determination of such changes may serve to establish earlier and specific therapeutic interventions for to the causative agent.

Of note, COVID-19 subjects without neurological symptoms did not display changes in ACE2 fragments, possibly because of the lack of viral replication within the brain. CoV patients with no encephalitis symptoms, who presented an overall increase in the levels of all ACE2 species, neither noticed imbalance between ACE2 fragments, which appears as a specific feature of CoV-Enceph patients. Indeed, inflammation in patients with or without COVID-19 could cause an increase in tissue ACE2 expression, as well as neuronal release, as revealed by an increase in soluble full-length species, and also T-tau and NfL levels [30]. A recent study reported an upregulation of brain ACE2 in certain COVID-19 patients, with the highest expression observed in the patients with the most severe neurological symptoms [31]. In this regard, soluble ACE2 levels have been found to be increased in several inflammatory processes, including acute lung injury [32], and ACE2 expression is responsive to inflammatory signaling and can be upregulated by viral infections or interferon treatment [33]. Several analyses suggest that ACE2 is upregulated during acute viral infection [34–36]. Thus, although the expression of ACE2 mRNA could be downregulated in the initial stage of SARS-CoV-2 infection, an upregulation occurs in later stages [37], perhaps as a consequence of massive inflammation. Hence, ACE2 expression is expected to be increased in SARS-CoV-2–infected tissues in association with an excessive immune/inflammatory response [38], but probably not in cases of minor infection/mild inflammatory response. Together these results support a link between ACE2 and inflammation that is not exclusive to COVID-19, indicating that levels of soluble full-length species of ACE2 may serve to evaluate an exacerbated inflammatory response during disease progression. In the few cases with severe neurological involvement, ACE2 expression might be dysregulated for different reasons. Of note, no patient had a positive result for CSF SARS-CoV-2 by RT-PCR, in line with a potential indirect rather than direct effect of the virus on brain-specific inflammatory-mediated processes [39, 40].

ADAM17/10 are the main sheddases for cleaving ACE2 ectodomain constitutively or in response to a stimulus [15, 41], but TMPRSS2 may also cleave ACE2 at a different site than ADAM17/10, thus potentially leading to differential outcomes [42]. Whether or not the particular increase in the shortest ACE2 fragment, triggered by SARS-CoV-2 infection, is a result of TMPRSS2 viral recruitment needs to be clarified in further studies.

Here, we revealed that TMPRSS2 was present in CSF as the zymogen and a fragment attributed to the active protease, and their levels were only increased in Enceph patients without COVID-19, showing also subtle differences in the balance of the different species identified by the electrophoresis/western blot analysis. The *TMPRSS2* gene encodes a so called isoform 2 protein [19], with a predicted molecular mass of approximately 55 kDa due to glycosylation, which is essential for zymogen activation and cell surface expression [43]. The resulting active fragment following zymogen cleavage has been described as an approximately 25-kDa species in cells overexpressing TMPRSS2 [44]. Regarding the presence of full-length species in CSF, it has been previously described in diverse human cell lines overexpressing TMPRSS2 that activation by zymogen cleavage occurs before reaching the cell surface, but in all cell extracts the more abundant species is the zymogen [43], thus most of the protein remains unprocessed. In any case, the finding of circulating TMPRSS2 full-length species was not surprising.

In CoV-Enceph, we failed to detect significant differences compared to controls for the levels of the 55-kDa zymogen or the 25-kDa fragment. However, we observed an interesting trend of an increased 25 kDa/55 kDa quotient, as the 25-kDa fragment displayed a nonsignificant increase, while the full-length 55-kDa species tended to decrease. However, in this case, this quotient did not serve to discriminate between Enceph patients with and without COVID-19, because in Enceph patients with increased TMPRSS2 the 25-kDa fragment was elevated in a higher proportion than that of the 55-kDa species. Despite the common feature in Enceph patients with and without COVID-19 regarding the imbalance between TMPRSS2 zymogen and the active fragment, the reason why CoV-Enceph patients do not present an overall increase in protein levels, as in Enceph patients, is unknown. Because the generation of a particular proteolytic fragment of ACE2 occurs in the presence of subtle changes in TMPRSS2 levels, it would be of interest to study levels of other proteases that can process ACE2, such as ADAM17/10 [42].

This study has several limitations that must be acknowledged. Most of the limitations are due to sample size and the analytical approach. In particular, the size of the COVID-19 group without encephalitis-related neurological symptoms is very small, because lumbar puncture was performed in these few cases only for ruling out atypical headache, but it is an invasive procedure indicated only for neurological symptoms. However, the cohort is the largest published with extensive CSF markers available so far [7]. The limited sample size also prevents further analysis by bioinformatics tools, which would be valuable in the future to decipher which changes in ACE2/TMPRSS2 are related to inflammatory mechanisms and which to the brain infection. Moreover, CSF levels were not compared with analysis of brain tissue, and we assumed, but did not determine, that ACE2/TMPRSS2 full-length species reflect tissue abundance. Furthermore, we conducted the analysis by western blotting, which has clear disadvantages in comparison to approaches for quantitative analysis such as enzyme-linked immunosorbent assay (ELISA); however, these methods do not detect subtle changes in specific species, reflecting specific outcomes of processing. Moreover, we have experience using fluorescent-based imaging for many proteins and particularly for ACE2 analysis [17, 18, 45]. The definition of the quotient between the 80-kDa SARS-CoV-2-related fragment and the 85-kDa fragment could result in an easy-to-use tool to reduce the quantitative limitations of western blotting, and increase the reliability and confidence of the neurochemical diagnosis. Moreover, many factors such as age, sex, and smoking, could potentially influence plasma ACE2 levels [46], as well as comorbidities or treatments that course with altered ACE2 levels [47]. The association of the shortest ACE2 fragment with SARS-CoV-2 cell infection could serve to prevent the impact of patient heterogeneity. Indeed, the severity of COVID-19 could also confound conclusions if only total ACE2 levels are considered. Thus, in patients with moderate disease or in the early phase of SARS-CoV-2 infection, ACE2 levels may appear unchanged given the decrease in full-length species from shedding, while inflammation, independent of tissue infection, can cause an increase in ACE2 expression [47], with an overall increase in all ACE2 species. Again, the quotient between the shortest ACE2 fragment and other species could serve to discriminate the occurrence of effective SARS-CoV-2 cellular penetrance. The development of specific biochemical methods for the determination of the SARS-CoV-2 related ACE2 fragment in particular fluids could serve in clinical practice to evaluate the occurrence of SARS-CoV-2 infection in particular tissues.

In conclusion, our study provides direct evidence of SARS-CoV-2–induced ACE2 expression and processing in the brain of COVID-19 patients presenting with encephalitis. In particular, the increase of the shortest ACE2 fragment related to the CoV-Enceph condition, and reflected in an altered 80 kDa/85 kDa quotient, could reveal the enhanced cleavage of the receptor triggered by SARS-CoV-2 and may predict occurrence of the rare encephalitis complication. Our previous report on plasma [17] and the present finding in CSF validate that monitoring increased levels of the shortest ACE2 fragment could serve for monitoring of viral penetrance in particular organs and the complications associated with COVID-19. Despite having related constraints, the consistent findings of the increased generation of a specific ACE2 fragment among SARS-CoV-2–infected patients bolster the robustness of this study's conclusions. Further studies, particularly follow-up studies to examine whether ACE2 fragments levels are restored in patients after recovery, are needed, as well in vitro studies designed to understand the molecular mechanisms underpinning this severe neurological complication of COVID-19.

## Supplementary Material

jiaf093_Supplementary_Data

## Supplementary Data

Supplementary materials are available at *The Journal of Infectious Diseases* online (http://jid.oxfordjournals.org/). Supplementary materials consist of data provided by the author that are published to benefit the reader. The posted materials are not copyedited. The contents of all supplementary data are the sole responsibility of the authors. Questions or messages regarding errors should be addressed to the author.

## Notes

***Acknowledgments*.** The authors thank the study participants who took part in this research and the neurological teams involved in the COVID-19 pandemic management. We thank Sergio Fuster and Carmen Marquez for their technical assistance.

***Author contribution.*** M. P. L., A. Pi., and J. S.-V. contributed to design of the work, acquisition, analysis, and interpretation of data. M. P. L., M.-S. G.-A., C. A.-G., C. Tr., C. To., V. Q., D. A., V. C., I. V., F. C., V. D. G., S. M., S. F., G. Z., N. J. A., H. Z., K. B., and A. Pa. acquired data. M. P. L., M.-S. G.-A., C. A.-G., C. Tr., A. P., and J. S.-V. performed data analysis and statistics. M. P. L., M.-S. G.-A., C. Tr., A. Pa., A. Pi., and J. S.-V. drafted and substantively revised the manuscript. All the authors revised and approved the submitted version.

***Data availability.*** The datasets used and analyzed during the current study are available from the corresponding author on reasonable request.

***Financial support.*** This work was supported by Fondo de Investigaciones Sanitarias (grant number PI22-01329 to J. S.-V.) cofunded by the Fondo Europeo de Desarrollo Regional Investing in your Future; Centro de Investigación Biomédica en Red Sobre Enfermedades Neurodegenerativas (CIBERNED), Instituto de Salud Carlos III (to J. S.-V.); Instituto de Investigación Sanitaria y Biomédica de Alicante (to J. S.-V.); Direcció General de Ciència i Investigació, Generalitat Valenciana (grant number AICO/2021/308 to J. S.-V.); Conselleria d'Innovació, Universitats, Ciència i Societat Digital (grant number INVEST-2023-157 to J. S.-V.) cofunded by the Fondo Europeo de Desarrollo Regional Investing in your Future, Generalitat Valenciana; Spanish Ministerio de Ciencia, Innovación y Universidades, Agencia Estatal de Investigación (AEI/10.13039/501100011033), through the Severo Ochoa Programme for Centres of Excellence in R&D (grant number CEX2021-001165-S to J. S.-V.); the Generalitat Valenciana ACIF/2019 (to M. P. L.); the Italian Ministry of University and Research (to C. Tr.); European Union Horizon 2020 Innovative Medicines Initiative IDEA-FAST (grant number ID853981 to V. Q., A. Pa., and A. Pi.); Italian Ministry of Health PRIN 2021 RePlast (to C. To.); Swedish Research Council (grant numbers 2023-00356, 2022-01018, 2019-02397 to H. Z. and 2017-00915 and 2022-00732 to K. B.); European Union Horizon 2020 (grant number 101053962 to H. Z.); Swedish State Support for Clinical Research (grant number ALFGBG-71320 to H. Z.); the Alzheimer Drug Discovery Foundation, United States (grant number 201809-2016862 to H. Z.); Alzheimer's Association Alzheimer's Disease Strategic Fund (grant numbers ADSF-21-831376-C, ADSF-21-831381-C, ADSF-21-831377-C, and ADSF-24-1284328-C to H. Z., SG-23-1038904 QC to K. B., and ZEN-21-848495 Zenith Award to K. B.); the Bluefield Project; Cure Alzheimer's Fund; the Olav Thon Foundation; the Erling-Persson Family Foundation; Stiftelsen för Gamla Tjänarinnor; Hjärnfonden (grant number FO2022-0270 to H. Z., and FO2017-0243 and ALZ2022-0006 to K. B.); European Union Horizon 2020 Marie Skłodowska-Curie Actions MIRIADE project (grant number 860197 to H. Z.); European Union Joint Programme—Neurodegenerative Disease Research (grant number JPND2021-00694 to H. Z. and JPND2019-466-236 to K. B.); the National Institute for Health and Care Research University College London Hospitals Biomedical Research Centre, and the UK Dementia Research Institute at UCL (grant number UKDRI-1003 to H. Z.); Swedish Government and the County Councils Avtal om Läkarutbildning och Forskning Agreement (grant numbers ALFGBG-715986 and ALFGBG-965240 to K. B.); Italian Ministry of University and Research (grant number 2017MYJ5TH PRIN COCOON and 20202THZAW PRIN 2021 RePlast to A. Pa. and A. Pi.); Italian Ministry of Health (grant numbers RF-2018-12366209, RF-2019-12369272, and PNRR-Health PNRR-MAD-2022-12376110 to A. Pa. and A. Pi.); Airalzh Foundation Life-Bio grant (grant number AGYR2021 to A. Pi.); and the LIMPE-DISMOV Foundation Segala Grant 2021 (to A. Pi.).
