# Supplementary material for: Increased Cerebrospinal Fluid Angiotensin-Converting Enzyme 2 Fragments as a Read-Out of Brain Infection in Patients With COVID-19 Encephalopathy
Source: J Infect Dis. 2025 Feb 26;231(5):e929–40. doi: 10.1093/infdis/jiaf093 (PMC12128074; doi:10.1093/infdis/jiaf093)
Supplement: jiaf093_Supplementary_Data [file jiaf093_supplementary_data.docx]

**Supplemental Information**

**Methods**

**Patient cohorts**

The study was carried out in accordance with the Declaration of Helsinki. The Institutional Ethical Standards Committee on human experimentation at Brescia University Hospital (Italy) provided approval for the study (NP 4067), as well as the Ethical Committee of the Universidad Miguel Hernandez (IN.JSV.03.20) (Spain).

The case definition included any person aged > 18 admitted to hospital with altered mental status lasting ≥ 24 hours, and the presence of two or more of the following criteria: i) generalized or partial seizures not fully attributable to a pre-existing epilepsy, ii) new onset of focal neurologic findings. iii) CSF white blood cell count ≥ 5/cubic mm, iv) abnormality of brain parenchyma on neuroimaging suggestive of encephalitis that was either new from prior studies or appears acute in onset, v) abnormality on electroencephalography consistent with encephalitis. To increase specificity of COVID-19 related encephalitis, fever was not considered as a supportive feature for encephalitis, as indicated by standard criteria [1], as it is highly prevalent in COVID-19 disease. See flowchart summarizing cohort selection.

Flowchart summarizing cohort selection.


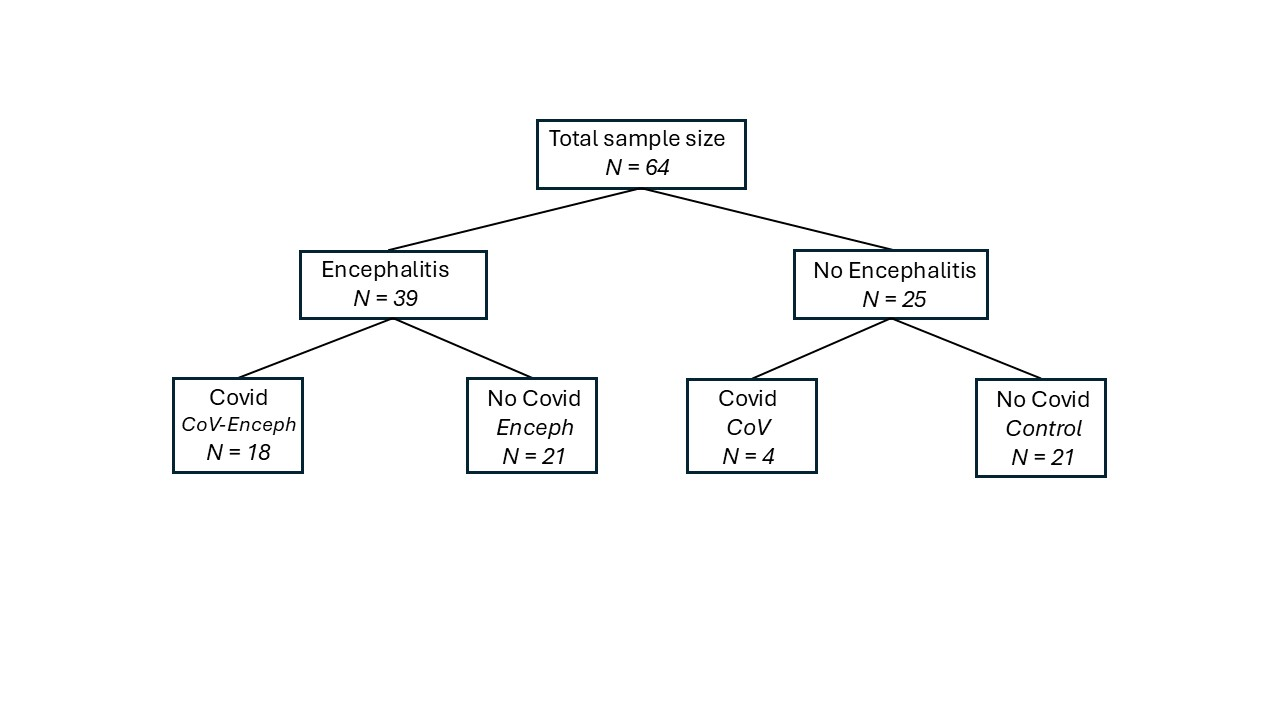


**Encephalitis assessment and diagnosis**

Each subject underwent brain magnetic resonance imaging, standard electroencephalography (EEG), thyroid function and antibodies (anti-thyroglobulin, anti-thyroid peroxidase), IgM and IgG for *Borrelia burgdorferi*. CSF viral screening included herpes simplex virus (HSV-1, HSV-2, HSV-6, HSV-8, CMV, Epstein-Barr virus, varicella zoster virus), adenovirus and enterovirus. All COVID-19 encephalitis resulted negative at the standard immunological screening, which included antibodies against NMDAR, LGI1, CASPR2, GABAbR, AMPAR, DPPX, Ri, Yo, Ma2, CV2, Hu, amphiphysin, titin (Euroline and Mosaic kit, Euroimmun, Luebeck) and MOG (live cell-based assay) at the Neurology Unit, Department of Neurosciences, Biomedicine and Movement Sciences, University of Verona, Italy. In COVID-19 disease cases, laboratory confirmation of SARS-CoV-2 infection was carried out by RT-PCR procedure on throat swab and nasopharyngeal specimens and CSF in all patients [2].

**CSF biomarkers for inflammation, brain injury and neurodegeneration**

Inflammatory and neuronal/glial markers were determined at the Clinical Neurochemistry Laboratory at Sahlgrenska University Hospital (Mölndal, Sweden), as previously reported [40]. CSF cytokine concentrations (IL-6, IL-8, TNF-α, IL-1β) were measured using a Mesoscale Discovery multiplexed immunoassay (Rockville, MD). CSF T-tau and P-tau181 concentrations were measured by Lumipulse (Fujirebio, Ghent, Belgium). CSF Aβ38, Aβ40 and Aβ42 were determined using the MSD Triplex Assay (MSD, Rockville, MD. CSF NfL and GFAP concentrations were measured using in-house enzyme-linked immunosorbent assays [3,4]. CSF sTREM2 concentration was measured using an in-house immunoassay with electrochemiluminescent detection, as previously described in detail [5]. CSF YKL-40 concentration was measured using the Human Chitinase 3-like 1 Quantikine kit (R&D Systems, Minneapolis, MN). All analyses were performed by board-certified laboratory technicians who were blinded to clinical data.

**Determination of ACE2 and TMPRSS2 in CSF samples by quantitative fluorescent western blotting**

At enrollment, 3 mL of CSF from each participant were collected, centrifuged and firstly processed for standard biochemical analyses. Two mL of CSF were stored in cryotubes at −80°C before testing. ACE2 and TMPRSS2 species were detected by fluorescent-based imaging after sodium dodecyl sulphate-polyacrylamide gel electrophoresis (SDS-PAGE) and western blotting. This technique provides a wider linear dynamic range than chemiluminescent detection, including a greater upper linear range of detection [6].

CSF samples were heated in reducing Laemmli SDS sample buffer (Thermo Scientific^TM^) for 7 min at 70°C. CSF samples (20 μL loaded) were then resolved on 7.5% for ACE2, and 12% for TMPRSS2, SDS-PAGE gels (Mini-PROTEAN® TGX™ Precast Gels; Bio-Rad) and transferred to 0.45 μm nitrocellulose membranes (Bio-Rad). Then, the membrane was blocked with Odyssey Blocking Buffer (PBS) and incubated with anti-ACE2 or anti-TMPRSS2 antibodies as described. Finally, blots were washed and incubated with the appropriate conjugated secondary antibodies (IRDye 800CW donkey anti-goat, IRDye 800CW goat anti-mouse, IRDye 680RD goat anti-mouse and IRDye 680 RD goat anti-rabbit, LI-COR Biosciences) and imaged on an Odyssey Clx Infrared Imaging System (LI-COR Biosciences). For quantitative analysis of ACE2 all blots were incubated with the AF933 antibody, and TMPRSS2 with 14437-1-AP antibody, which resolve all soluble species. An aliquot of the same control CSF sample was included in all the blots to normalize the immunoreactive signal between blots. All samples were analysed at least in duplicate. Band intensities were analysed using LI-COR software (Image Studio Lite).

**Data availability**

All clinical and CSF analyses data are available from authors upon reasonable request.

**References**

1. Venkatesan A, Tunkel AR, Bloch KC, et al. Case definitions, diagnostic algorithms, and priorities in encephalitis: consensus statement of the international encephalitis consortium. Clin Infect Dis Off Publ Infect Dis Soc Am **2013**; 57:1114–1128.

2. Pilotto A, Masciocchi S, Volonghi I, et al. Clinical Presentation and Outcomes of Severe Acute Respiratory Syndrome Coronavirus 2-Related Encephalitis: The ENCOVID Multicenter Study. J Infect Dis **2021**; 223:28–37.

3. Rosengren LE, Wikkelsø C, Hagberg L. A sensitive ELISA for glial fibrillary acidic protein: application in CSF of adults. J Neurosci Methods **1994**; 51:197–204.

4. Gaetani L, Höglund K, Parnetti L, et al. A new enzyme-linked immunosorbent assay for neurofilament light in cerebrospinal fluid: analytical validation and clinical evaluation. Alzheimers Res Ther **2018**; 10:8.

5. Banerjee G, Ambler G, Keshavan A, et al. Cerebrospinal Fluid Biomarkers in Cerebral Amyloid Angiopathy. J Alzheimers Dis **2020**; 74:1189–1201.

6. Mathews ST, Plaisance EP, Kim T. Imaging systems for westerns: chemiluminescence vs. infrared detection. Methods Mol Biol Clifton NJ **2009**; 536:499–513.
